# The Association Between Irritable Bowel Syndrome and Generalized Anxiety Disorder and Influencing Factors: A Mediation Mendelian Randomization Study

**DOI:** 10.1002/fsn3.71525

**Published:** 2026-02-10

**Authors:** Weili Yang, Yongxi Wang, Shasha Wang, Hongbing Zhai, Jun Che, Xin Wang, Yafeng Yang, Zebo Jia

**Affiliations:** ^1^ School of Public Health, Department of Applied Psychology Shaanxi University of Chinese Medicine Xianyang Shaanxi China; ^2^ Department of Gastroenterology Xianyang Central Hospital Xianyang Shaanxi China; ^3^ Department of Nutrition Xianyang Central Hospital Xianyang Shaanxi China

**Keywords:** generalized anxiety disorder, gut microbiota, immune, irritable bowel syndrome, mediation analysis, Mendelian randomization

## Abstract

Previous studies showed that irritable bowel syndrome (IBS) and generalized anxiety disorder (GAD) share a common pathogenic mechanism. However, research on links between immune cells, plasma metabolites, inflammatory factors, and gut microbiota and these diseases remains limited. This study aimed to probe causal relationships between these factors and IBS/GAD using Mendelian randomization (MR) analysis. Among factors associated with IBS, 25 gut microbial taxa, 103 plasma metabolites, 7 inflammatory factors, and 42 immune cell characteristics had causal relationships with IBS. Among factors associated with GAD, 35 gut microbial taxa, 72 plasma metabolites, 6 inflammatory factors, and 43 immune cell characteristics had causal links to GAD. IBS was appointed as a risk factor for GAD [odds ratio (OR) = 1.328; *p* < 0.001]. Mediation analysis showed that IBS acted as a mediator, modulating the effects of 1 immune cell, 1 gut microbiota, and 2 plasma metabolites on GAD. IBS attenuated the protective effects of “*Bilophila*” on GAD onset (13.30%). This study respectively revealed the potential causal roles of multiple factors in IBS and GAD, as well as the causal relationship between IBS and GAD. Additionally, the mediating role of IBS was unveiled, delivering fresh perspectives on the pathogenesis of IBS and GAD.

## Graphical abstract

Based on Mendelian randomization analysis, this study established a significant causal effect of irritable bowel syndrome (IBS) on increasing generalized anxiety disorder (GAD) risk (odds ratio = 1.328; *p* < 0.001) and identified dozens of gut microbial taxa, plasma metabolites, inflammatory factors, and immune cell characteristics with potential causal relationships to either IBS or GAD. Critically, mediation analysis revealed IBS acts as an intermediary, attenuating protective effects—such as a 13.30% reduction for the gut genus ‘Bilophila’ against GAD—while also mediating effects of specific immune cells and metabolites, providing novel mechanistic insights into both conditions.

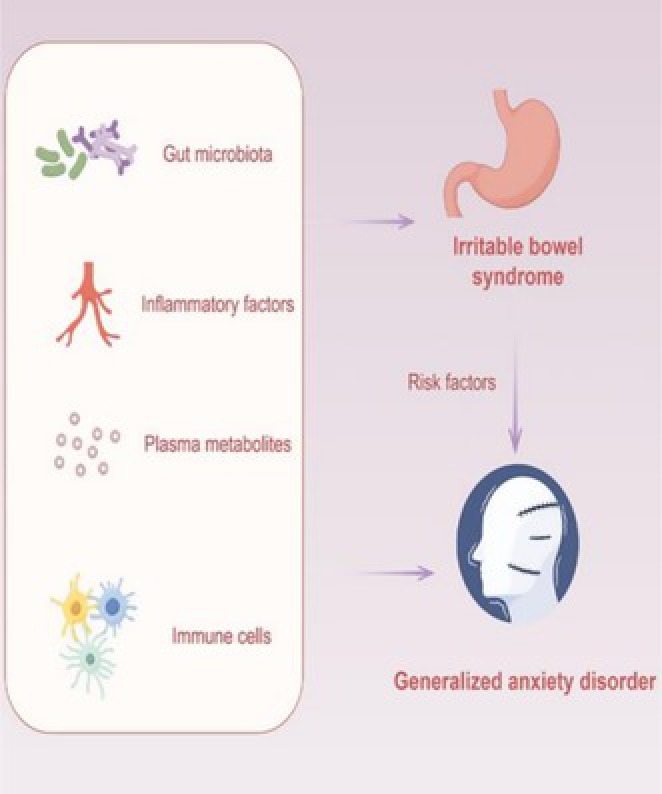

## 1 Introduction

Irritable bowel syndrome (IBS) is a prevalent chronic functional gastrointestinal disorder, marked by recurrent abdominal pain or discomfort and alterations in bowel habits (Y. Chen et al. 2024). Epidemiological data reveal that IBS affects 10%–20% of the global population, with prevalence rates continuing to rise (Staudacher et al. 2023). Generalized anxiety disorder (GAD) is a serious mental illness characterized by excessive and uncontrollable worry as well as persistent psychological and physical tension (Baldaçara et al. 2024; Y. Xue et al. 2025). The research by Chris Eijsbouts et al. (2021) indicates that there are common pathogenic pathways that can affect the occurrence of IBS and anxiety. Notably, clinical investigations have revealed that approximately 32% of IBS patients also manifest symptoms of GAD (Mayer et al. 2001). This indicates that IBS and GAD may both originate from a more upstream and fundamental biological disorder, and IBS may be a risk factor for GAD.

Research into the pathogenesis of IBS has traditionally focused on areas such as gastrointestinal motility abnormalities, heightened intestinal sensitivity, dysregulation of the brain–gut axis, and psychosocial influences (Kellow and Phillips 1987; Kerckhoffs et al. 2009; Mayer and Tillisch 2011; J. Rogers et al. 1989). The significance of immune‐inflammatory responses in IBS has also gained prominence (Ohman and Simrén 2010). Evidence suggests that IBS may involve an activated adaptive immune response, with increased epithelial barrier permeability and gut microbiota dysbiosis potentially leading to enhanced activation of the intestinal immune system (Ohman and Simrén 2010). Recent findings have correlated serum levels of tumor necrosis factor (TNF)‐α and interleukin (IL)‐17 with the severity of discomfort and symptoms in IBS patients (Choghakhori et al. 2017). Moreover, metabolomic research has highlighted the pivotal role of various metabolites in IBS pathophysiology, implicating them in crucial biological processes, including signal transduction and immune modulation (Han et al. 2022). It can be seen that immune cells, plasma metabolites, inflammatory factors, and gut microbiota play an important role in IBS.

The gut microbiota has been recognized as a significant factor in the body's physiological response to stress (Wiley et al. 2017), with its modulation exerting neurobiological effects (Westfall and Pasinetti 2019). Studies have documented gut dysbiosis in GAD patients, positing the microbiome as a novel therapeutic and preventive target for GAD (Jiang et al. 2018). Alterations in immune cell functionality have been shown to influence mood and anxiety states through effects on neurotransmitter systems (Won and Kim 2020). Investigations into the gut–brain axis further substantiate the role of immune system dysregulation as a potential mechanism underlying anxiety disorders (Dai et al. 2025). Metabolomic analyses have linked abnormalities in metabolic pathways involving phenylalanine, tyrosine, and tryptophan to the etiology of anxiety disorders (Kui et al. 2022). Exposure to trauma and stress, including fear‐ and anxiety‐inducing stimuli, has been associated with hypothalamic–pituitary–adrenal (HPA) axis reactivity, immune system activation, and the secretion of pro‐inflammatory cytokines (Haroon et al. 2012). Additionally, reduced levels of anti‐inflammatory cytokines, such as IL‐2 and IL‐4, have been observed in GAD patients (Vieira et al. 2010).

While prior research has examined the contributions of immune cells, plasma metabolites, inflammatory factors, and gut microbiota to IBS/GAD, the precise interrelationships remain elusive. Given that immune cells, plasma metabolites, inflammatory factors, and the gut microbiota all play significant roles in the pathogenesis of IBS and GAD. Therefore, this study aims to separately explore the influence of factors such as immune cells and plasma metabolites on IBS/GAD. In addition, IBS patients also exhibit symptoms of GAD. Therefore, we also further explored the mediating role of IBS. Genetic epidemiology has made considerable use of Mendelian randomization (MR), a method of causal inference based on genetic variation (Sekula et al. 2016). MR offers advantages over conventional observational studies by utilizing genetic variants as instrumental variables (IVs) to mitigate confounding, thereby bolstering the robustness of causal inferences (Sanderson et al. 2022).

## 2 Materials and Methods

### 2.1 Data Collection

The genome‐wide association study (GWAS) data on plasma metabolites, immune cell characteristics, and gut microbiota could be obtained from the GWAS catalog (https://www.ebi.ac.uk/gwas/). The Canadian Longitudinal Study on Aging (CLSA) cohort provided GWAS data on 1400 plasma metabolites, covering 8091 individuals (GCST90199621–GCST90201020) (Y. Chen et al. 2023). The GWAS data on 731 immune cell characteristics (GCST0001391–GCST0002121) involved 3757 non‐overlapping European individuals (Orrù et al. 2020). The data on gut microbiota were derived from the Dutch Microbiome Project, which included 7738 participants and measured 207 microbial taxa and 205 metabolic pathways (GCST90027446–GCST90027857) (Lopera‐Maya et al. 2022). A study used the Olink Target Inflammation panel to measure 91 inflammation‐related circulating proteins, involving 11 cohorts and 14,824 European participants (Zhao et al. 2023). The data on IBS were downloaded from the IEU OpenGWAS project (https://gwas.mrcieu.ac.uk/). This ebi‐a‐GCsT90016564 dataset included 53,400 cases and 433,201 control samples of European ancestry, with a total of 9,739,966 single‐nucleotide polymorphisms (SNPs). The data on GAD (finngen_R12_F5_GAD) were obtained from FinnGen R12 (https://r12.finngen.fi/), including 7148 cases and 444,414 European control samples (Kurki et al. 2023).

### 2.2 Acquisition of IVs

The analysis strictly adhered to the MR reporting guidelines for observational studies (STROBE‐MR) (Table S1) (Skrivankova et al. 2021). The entire study was based on three fundamental assumptions: (1) There should be a significant connection between the exposure and the IVs chosen. (2) IVs ought to be unaffected by confounding variables. (3) IVs must only influence the result by exposure. To maximize the utility of the IVs, the following selection criteria were applied: When the exposure factors were immune cell characteristics, plasma metabolites, inflammatory factors, and gut microbiota, SNPs with *p* < 1 × 10^−5^ were chosen as IVs; whereas for IBS or GAD exposures, a more stringent criterion of *p* < 5 × 10^−6^ was adopted. The ieugwasr package (v 1.0.0) (Fan et al. 2024) was used to exclude SNPs with linkage disequilibrium, with parameters set at *r* ^2^ = 0.001 and kb = 10,000. Additionally, SNPs that were substantially linked to the exposure or result (*p* < 1 × 10^−5^) were excluded via the GWAS catalog to avoid potential horizontal pleiotropy. To assess the strength of each SNP as an IV, the F‐statistic was worked out, and SNPs with an F‐statistic < 10 were excluded. Subsequently, the harmonise_data function was utilized to integrate exposure and outcome data, ensuring consistent SNP effect directions. Finally, the steiger_filtering function was solicited to detect the direction of association, retaining only SNPs with associations from exposure to outcome.

### 2.3 MR Analysis

The study first employed MR analysis to systematically analyze the causal connection between immune cell characteristics, plasma metabolites, inflammatory factors, and gut microbiota with IBS and GAD. Additionally, the mediating effects of IBS on the associations between these multiple factors and GAD were thoroughly analyzed. In terms of analytical methods, the inverse variance weighted (IVW) model (Ding et al. 2023) was resorted to as the cardinal tool. To verify the robustness of the results, several supplementary methods were also employed, including MREgger (Burgess and Thompson 2017), weighted median (Bowden et al. 2016), simple mode (Zeng et al. 2023), and weighted mode (Zeng et al. 2023), to estimate causal effects from multiple perspectives. In mediation analysis, the total causal effect of the exposure on the outcome (c) was first calculated, followed by the causal effects of the mediator on the outcome (b) and the exposure on the mediator (a). The product a*b was then derived as the mediating effect, c‐a*b as the direct effect, and a*b/c as the proportion of the mediating effect. The standard errors of the mediating effect, direct effect, and mediating proportion were estimated using the delta method (Carter et al. 2019).

### 2.4 Statistical Analysis

All analyses were primarily based on the TwoSampleMR (v 0.6.0) (Hemani, Zheng, et al. 2018) and MRPRESSO packages (v 1.0) (Zhu et al. 2022). The MRPRESSO tool was operated to detect and correct for pleiotropy by identifying and removing potential outliers with *p <* 0.05, thereby enhancing the stoutness of the analysis results. Additionally, to ascertain whether there truly was horizontal pleiotropy throughout IVs and the result, the MR–Egger intercept (*p >* 0.05) was taken into account. To quantify the degree of heterogeneity, the *p*‐value derived from the Cochrane Q statistic was reckoned. Leave‐one‐out was also executed to evaluate whether the IVW estimate was overly influenced by individual SNPs (Hemani, Bowden, and Davey Smith 2018).

## 3 Result

### 3.1 Selection of SNPs

In the genetic studies of IBS and GAD, a total of 52 SNPs were included, with *F*‐values spanning from 20.863 to 36.993. In the reverse MR analysis, 18 SNPs were selected, with *F*‐values varying from 20.957 to 28.681 (Table S2). For the study of immune cell characteristics and IBS, 17,358 SNPs were used, while for the study with GAD, 17,564 SNPs were selected, with *F*‐values spanning from 19.548 to 2435.818 (Table S3). In the analysis of plasma metabolites and IBS, 32,868 SNPs were obtained; in the analysis with GAD, 33,335 SNPs were obtained, with *F*‐values fluctuating from 19.511 to 5309.700 (Table S4). In the investigation of gut microbiota and IBS, 4036 SNPs were obtained, while in the study with GAD, 3910 SNPs were obtained, with *F*‐values fluctuating from 19.512 to 61.116 (Table S5). Finally, in the study of inflammatory factors and IBS, 2503 SNPs were selected, while in the study with GAD, 2345 SNPs were obtained, with *F*‐values varying from 19.513 to 1477.144 (Table S6). These results demonstrate that the selected SNPs have strong statistical power, providing a reliable basis for subsequent causal relationship analyses.

### 3.2 Causal Associations of Multiple Factors With IBS

The underlying cause of IBS, an elaborate functional digestive condition, is still elusive. Recently, IBS has been increasingly recognized as being closely related to neurofunctional disorders of the brain–gut axis, with low‐grade inflammation and immunological changes also attracting attention in the context of IBS (Ohman and Simrén 2010). Additionally, the gut microbiome has been confirmed as a risk factor for IBS (Black and Ford 2020). Against this backdrop, the research project sought to methodically examine any possible link of causality between immune cells, plasma metabolites, inflammatory factors, and gut microbiota with IBS via MR analysis.

**FIGURE 1:**
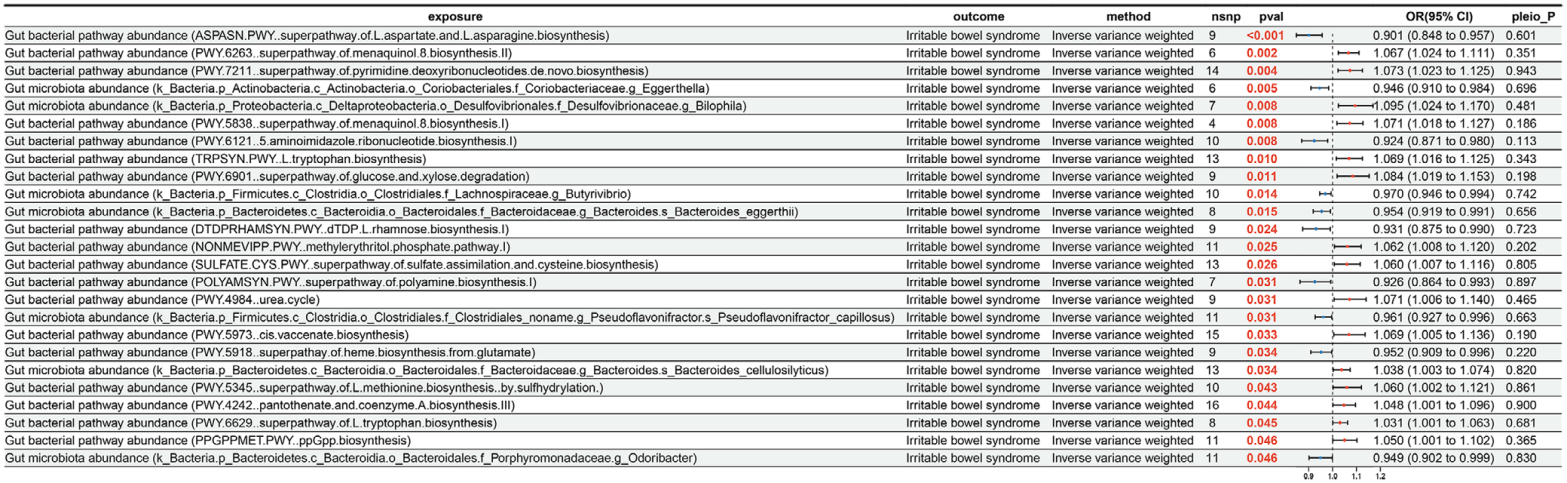
Results of Mendelian randomization (MR) analysis of 207 microbial taxa and 205 pathways on irritable bowel syndrome (IBS). CI, confidence interval; nsnp, number of single nucleotide polymorphisms; pleio_P, pleiotropy *p‐*value; pval, *p‐*value; or, odds ratio.

After traversing the causal relationships between 207 microbial taxa and 205 pathways and IBS, 25 significant causal relationships were revealed, with 10 considered protective factors and 15 as risk factors (Figure 1). Specifically, the “superpathway of L‐aspartate and L‐asparagine biosynthesis” (odds ratio [OR] = 0.901); 95% confidence interval (CI) = 0.848–0.957; *p* < 0.001 and “*Eggerthella*” (OR = 0.946; 95% CI = 0.910–0.984; *p* = 0.005) were found to have protective effects on IBS. In contrast, the “superpathway of pyrimidine deoxyribonucleotides de novo biosynthesis” (OR = 1.073; 95% CI = 1.023–1.125; *p* = 0.004) and “*Bilophila*” (OR = 1.095; 95% CI = 1.024–1.170; *p* = 0.008) were revealed as risk factors for IBS.

**FIGURE 2:**
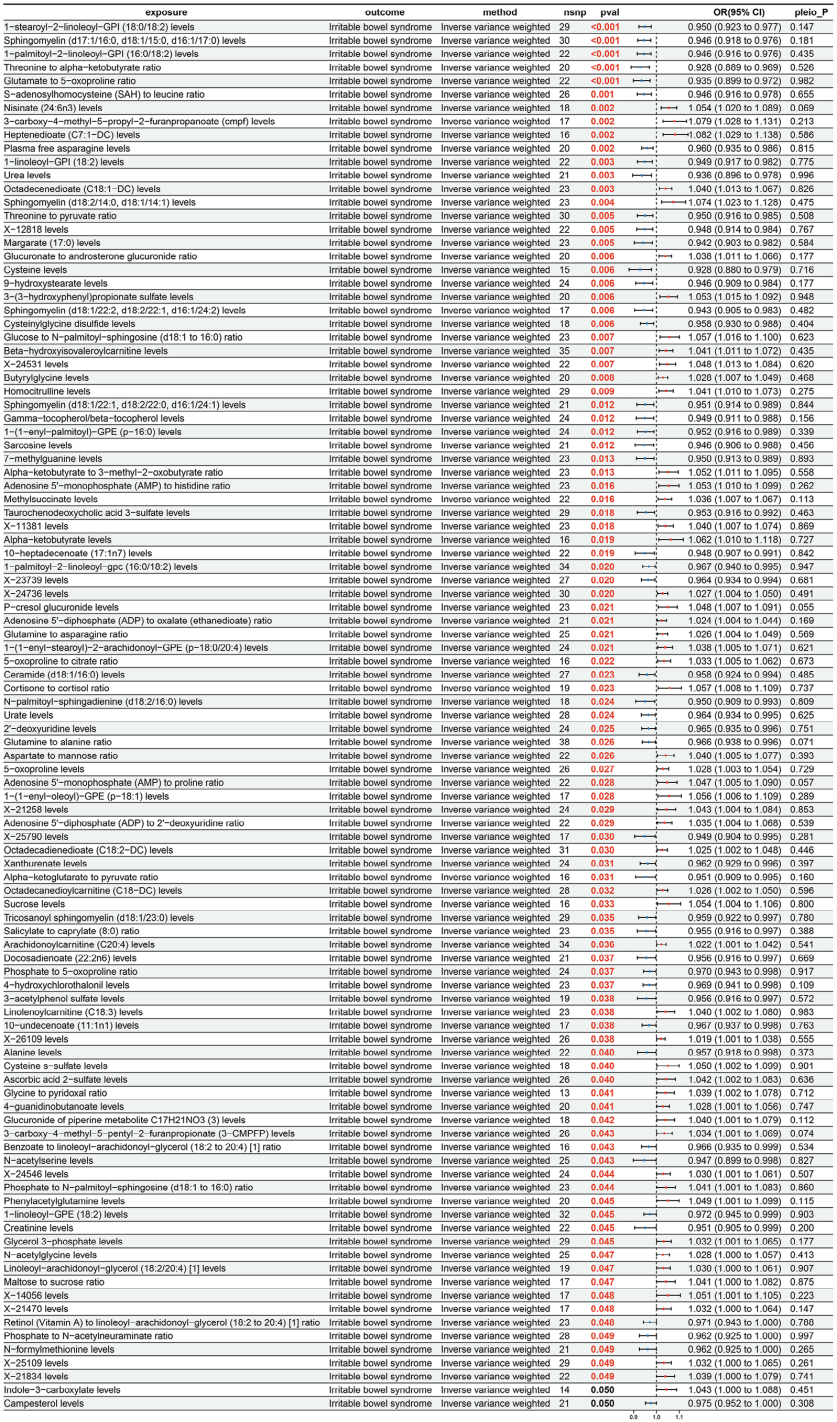
Results of MR analysis of plasma metabolites on IBS. CI, confidence interval; nsnp, number of single nucleotide polymorphisms; pleio_P, pleiotropy *p‐*value; pval, *p‐*value; or, odds ratio.

Following a thorough analysis of the putative causal links among 1400 plasma metabolites and IBS, 103 significant causal relationships were revealed, with 49 considered protective factors and 54 as risk factors (Figure 2). The IVW results showed that “Benzoate to linoleoyl‐arachidonoyl‐glycerol (18:2–20:4) [1] ratio” (OR = 0.966; 95% CI = 0.935–0.999; *p* = 0.043) and “S‐adenosylhomocysteine (SAH) to leucine ratio” (OR = 0.946; 95% CI = 0.916–0.978; *p* = 0.001) had protective effects on IBS. Conversely, “glucuronide of piperine metabolite C17H21NO3 (3) levels” (OR = 1.040; 95% CI = 1.001–1.079; *p* = 0.042) and “Adenosine 5′‐monophosphate (AMP) to histidine ratio” (OR = 1.053; 95% CI = 1.010–1.099; *p* = 0.016) were probed as risk factors for IBS.

**FIGURE 3:**
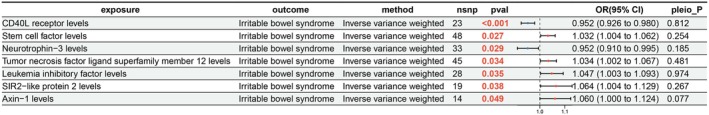
Results of MR analysis of inflammatory factors on IBS. CI, confidence interval; nsnp, number of single nucleotide polymorphisms; pleio_P, pleiotropy *p‐*value; pval, *p‐*value; or, odds ratio.

After exploring the potential causal relationships between 91 inflammatory factors and IBS, 7 significant causal links were revealed, with 2 considered protective variables and 5 as risk factors (Figure 3). The IVW results indicated that “tumor necrosis factor ligand superfamily member 12 levels” (OR = 1.034; 95% CI = 1.002–1.067; *p* = 0.034) and “leukemia inhibitory factor levels” (OR = 1.047; 95% CI = 1.003–1.093; *p* = 0.035) had detrimental effects on IBS. In contrast, “neurotrophin‐3 levels” (OR = 0.952; 95% CI = 0.910–0.995; *p* = 0.029) and “CD40L receptor levels” (OR = 0.952; 95% CI = 0.926–0.980; *p* = 0.001) were scrutinized as protective factors for IBS.

**FIGURE 4:**
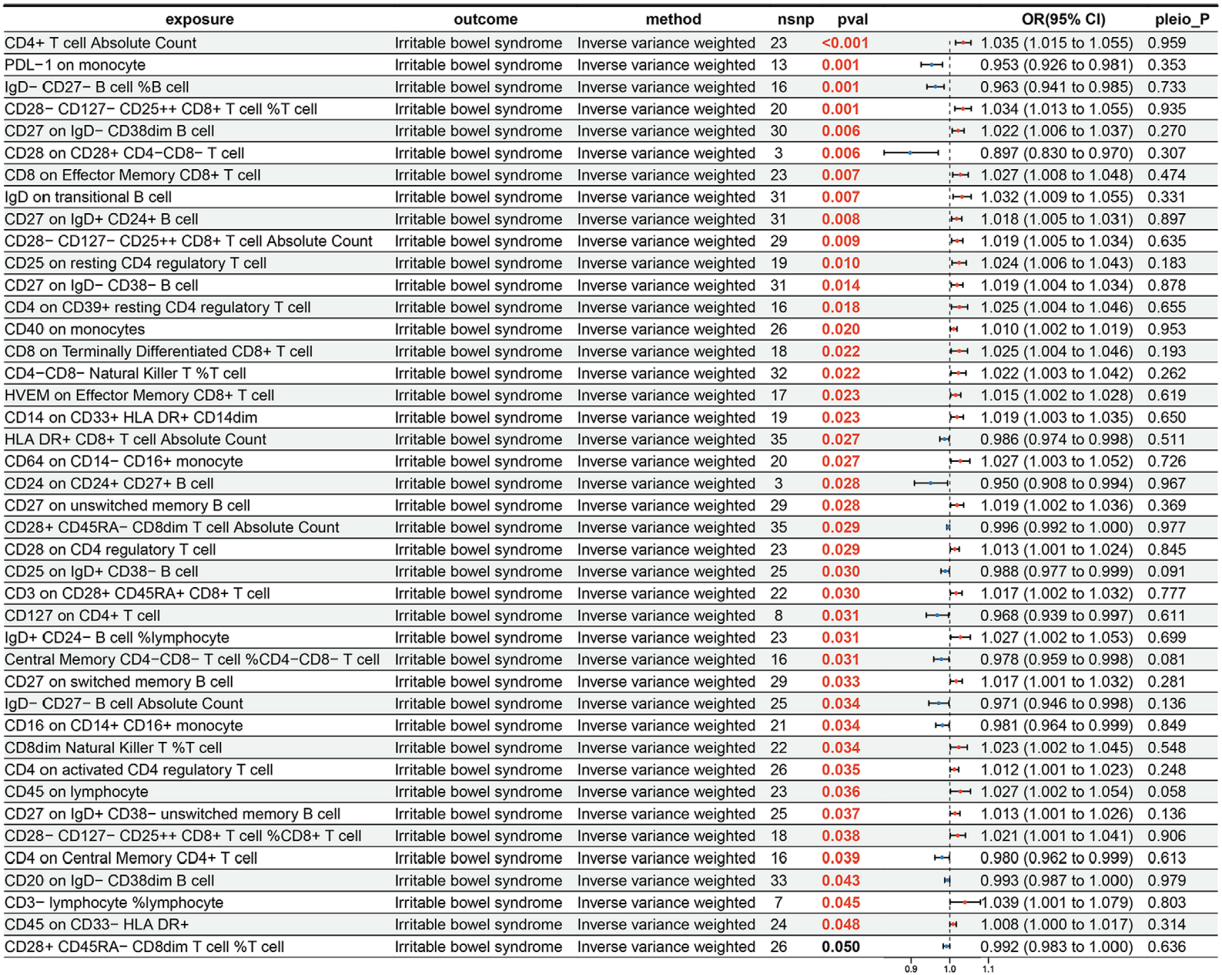
Results of MR analysis of immune cell characteristics on IBS. CI, confidence interval; nsnp, number of single nucleotide polymorphism; pleio_P, pleiotropy *p‐*value; pval, *p‐*value; or, odds ratio.

After an in‐depth exploration of the embryonic causal relationships among 731 immune cell characteristics and IBS, 42 significant causal relationships were revealed, with 14 considered protective factors and 28 as risk factors (Figure 4). Specifically, the IVW results showed that “central memory CD4^−^CD8^−^ T cell %CD4^−^CD8^−^ T cell” (OR = 0.978; 95% CI = 0.959–0.998; *p* = 0.031) and “CD4 on central memory CD4^+^ T cell” (OR = 0.980; 95% CI = 0.962–0.999; *p* = 0.039) had protective effects on IBS. Conversely, “CD8 on effector memory CD8^+^ T cell” (OR = 1.027; 95% CI = 1.008–1.048; *p* = 0.007) and “CD4 on CD39^+^ resting CD4 regulatory T cell” (OR = 1.025; 95% CI = 1.004–1.046; *p* = 0.018) were revealed as risk factors for IBS.

These results showcased the intricate causal links that exist within immune cells, plasma metabolites, inflammatory factors, and gut microbiota with IBS, granting unique viewpoints on the pathogenesis of IBS and further supporting the notion that IBS is a disease influenced by multiple factors.

### 3.3 Exploring the Causal Links of Multiple Factors With GAD

**FIGURE 5:**
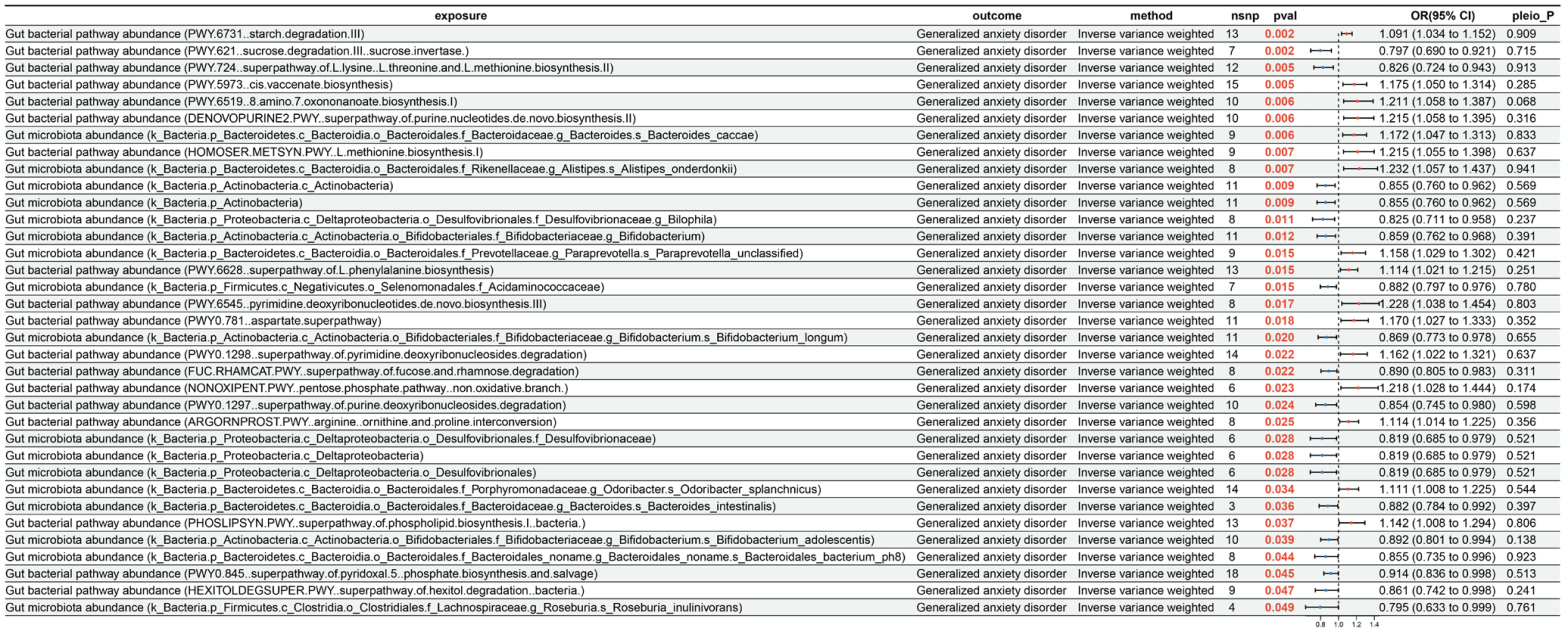
Results of MR analysis of 207 microbial taxa and 205 pathways on generalized anxiety disorder (GAD). CI, confidence interval; nsnp, number of single nucleotide polymorphism; pleio_P, pleiotropy *p‐*value; pval, *p‐*value; or, odds ratio.

Similarly, the causal links of the aforementioned factors with GAD were also investigated. After exploring the potential causal relationships between 207 microbial taxa and 205 pathways and GAD, 35 significant causal relationships were revealed, with 19 considered protective factors and 16 as risk factors (Figure 5). Specifically, “ *Roseburia inulinivorans* ” (OR = 0.795; 95% CI = 0.633–0.999; *p* = 0.049) and “sucrose degradation III (sucrose invertase)” (OR = 0.797; 95% CI = 0.690–0.921; *p* = 0.002) were found to have protective effects on GAD. In contrast, “ *Bacteroides caccae* ” (OR = 1.172; 95% CI = 1.047–1.313; *p* = 0.006) and “superpathway of pyrimidine deoxyribonucleosides degradation” (OR = 1.162; 95% CI = 1.022–1.321; *p* = 0.022) were scrutinized as risk factors for GAD.

**FIGURE 6:**
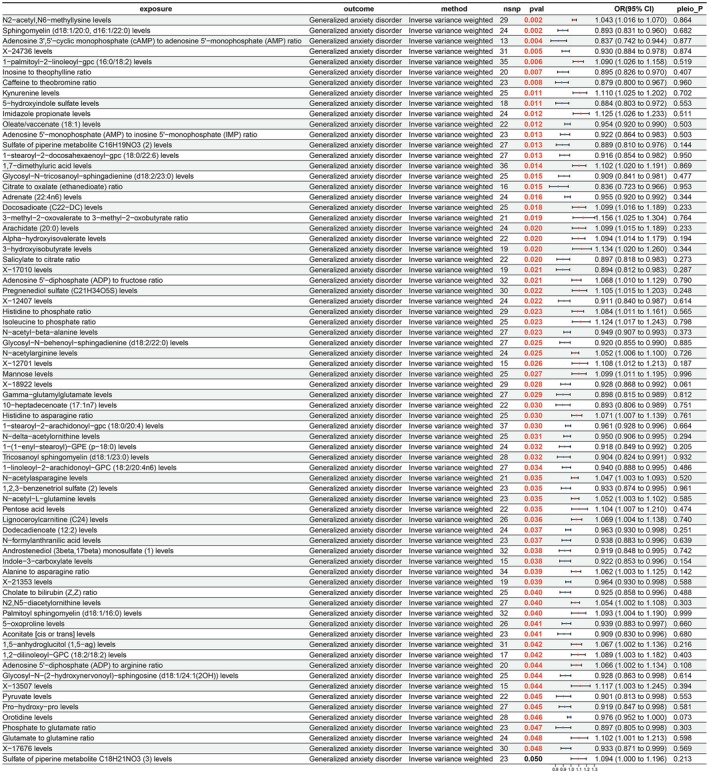
Results of MR analysis of plasma metabolites on GAD. CI, confidence interval; nsnp, number of single nucleotide polymorphisms; pleio_P, pleiotropy *p‐*value; pval, *p‐*value; or, odds ratio.

After exploring the potential causal relationships between 1400 plasma metabolites and GAD, 72 significant causal relationships were revealed, with 41 considered protective factors and 31 as risk factors (Figure 6). The IVW results showed that “AMP to inosine 5′‐monophosphate (IMP) ratio” (OR = 0.922; 95% CI = 0.864–0.983; *p* = 0.013) and “Adenosine 3′,5′‐cyclic monophosphate (cAMP) to AMP ratio” (OR = 0.837; 95% CI = 0.742–0.944; *p* = 0.004) had protective effects on GAD. Conversely, “glutamate to glutamine ratio” (OR = 1.102; 95% CI = 1.001–1.213; *p* = 0.048) and “alanine to asparagine ratio” (OR = 1.062; 95% CI = 1.003–1.125; *p* = 0.039) were scrutinized as risk factors for GAD.

**FIGURE 7:**
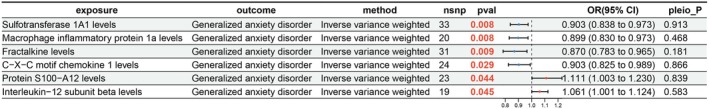
Results of MR analysis of inflammatory factors on GAD. CI, confidence interval; nsnp, number of single nucleotide polymorphisms; pleio_P, pleiotropy *p‐*value; pval, *p‐*value; or, odds ratio.

After exploring the potential causal relationships between 91 inflammatory factors and GAD, 6 significant causal relationships were revealed, with 4 considered protective factors and 2 as risk factors (Figure 7). For example, “protein S100‐A12 levels” (OR = 1.111; 95% CI = 1.003–1.230; *p* = 0.044) and “IL‐12 subunit beta levels” (OR = 1.061; 95% CI = 1.001–1.124; *p* = 0.045) were found to have detrimental effects on GAD. In contrast, “macrophage inflammatory protein 1a levels” (OR = 0.899; 95% CI = 0.830–0.973; *p* = 0.008) and “C‐X‐C motif chemokine 1 levels” (OR = 0.903; 95% CI = 0.825–0.989; *p* = 0.029) were scrutinized as protective factors for GAD.

**FIGURE 8:**
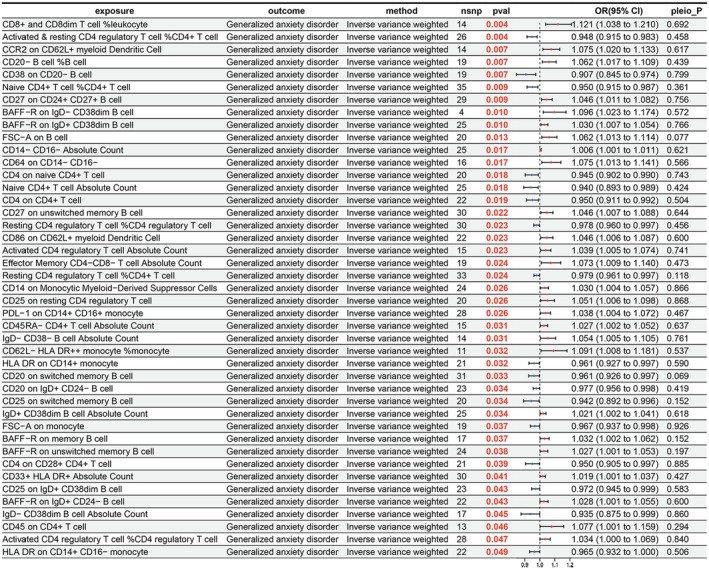
Results of MR analysis of immune cell characteristics on GAD. CI, confidence interval; nsnp, number of single nucleotide polymorphisms; pleio_P, pleiotropy *p‐*value; pval, *p‐*value; or, odds ratio.

After scouting the probable causal relationships among 731 immune cell characteristics and GAD, 43 significant causal relationships were revealed, with 17 considered protective factors and 26 as risk factors (Figure 8). Specifically, the IVW results showed that “activated & resting CD4 regulatory T cell %CD4^+^ T cell” (OR = 0.948; 95% CI = 0.915–0.983; *p* = 0.004) and “CD38 on CD20^−^ B cell” (OR = 0.907; 95% CI = 0.845–0.974; *p* = 0.007) had protective effects on GAD. Conversely, “CD14 on monocytic myeloid‐derived suppressor cells” (OR = 1.030; 95% CI = 1.004–1.057; *p* = 0.026) and “effector memory CD4^−^CD8^−^ T cell absolute count” (OR = 1.073; 95% CI = 1.009–1.140; *p* = 0.024) were scrutinized as risk factors for GAD.

### 3.4 Determining IBS as Risk Factor for GAD

Studies have shown that individuals with IBS commonly exhibit typical symptoms of GAD (Goodarzi and Rotter 2020). To further investigate the causal association between IBS and GAD, the MR method was employed. The IVW results indicated that IBS was a risk factor for GAD (OR = 1.328; 95% CI = 1.167–1.510; *p* < 0.001, false discovery rate [FDR] < 0.001) (Figure S1, Table S7). However, the reverse MR analysis did not demonstrate a causal link between GAD and IBS. This finding highlighted the complexity of the relationship between IBS and GAD and suggested that further exploration of other potential biological pathways or mediating factors was needed to fully understand the mechanisms of interaction between these two diseases.

### 3.5 Mediation Analysis Reveals Potential Associations Between IBS and GAD

**FIGURE 9:**
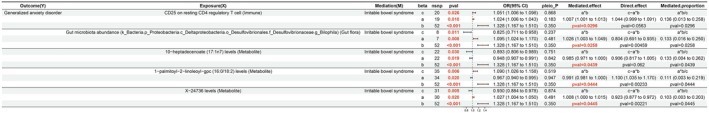
Results of mediation analysis of immune cell characteristic, gut microbiota, plasma metabolites via IBS for GAD. a, the effect of exposure on the mediator; b, the effect of mediator on the outcome; c, total causal effect of the exposure on the outcome; CI, confidence interval; nsnp, number of single nucleotide polymorphisms; pleio_P, pleiotropy *p‐*value; pval, *p‐*value; or, odds ratio.

To explore the complex links between IBS and GAD, mediation analysis was conducted. The results showed that when IBS acted as a mediating factor, 5 significant associations were revealed, including 1 immune factor, 1 gut microbiota, and 3 plasma metabolites that mediated the effects on GAD through IBS (Figure 9). IBS attenuated the protective effects of “*Bilophila*” (13.30%) and “X‐24736 levels” (10.30%) on GAD onset. This suggested that the protective effects of these factors on GAD were partially weakened through the mediating role of IBS. However, the specific nature of X‐24736 remains unclear at present. Conversely, IBS attenuated the detrimental effects of “1‐palmitoyl‐2‐linoleoyl‐gpc (16:0/18:2) levels” (11.10%) on GAD onset. In addition, IBS enhanced the risk effect of “CD25 on resting CD4 regulatory T cell” (13.60%) on GAD, as well as the protective effect of “10‐heptadecenoate (17:1n7) levels” (13.30%) on GAD. These findings indicated that IBS not only directly influenced the occurrence of GAD but also acted as a mediating factor, modulating the effects of immune cells, gut microbiota, and plasma metabolites on GAD. The existence of such mediating effects further revealed the complex links between IBS and GAD, suggesting that the relationship between the two was not merely a direct causal one but also involved indirect influences through multiple biological pathways.

### 3.6 Sensitivity Analysis

To ensure the robustness and reliability of the results, a variety of methods were employed. MR–Egger and MR‐PRESSO failed to pinpoint any hypothetical pleiotropy (Table S8). Faced with heterogeneity, we settled on a random‐effects model prior to the study to avoid bias caused by heterogeneity (Table S8). By sequentially excluding individual SNPs and re‐analyzing the remaining SNPs, we encountered that outcomes were not dramatically modified by the removal of a single SNP. These findings provided strong support for the results.

## 4 Discussion

IBS is a chronic disorder that is closely associated with significant psychological distress and psychiatric comorbidities. Patients with IBS often experience psychiatric symptoms such as anxiety, depression, and even suicidal ideation, which can severely impair their quality of life (Canavan et al. 2014). Research has demonstrated a high prevalence of psychiatric comorbidities in IBS, particularly depression and anxiety, which are present in up to 23% of IBS cases (Black and Ford 2020; Zamani et al. 2019). Additionally, the comorbidity of IBS with GAD has garnered considerable attention (Kaplan et al. 2014). The pathogenesis of IBS is multifactorial, encompassing changes in metabolite levels, gut microbiota dysbiosis, abnormal intestinal immune function, disruption of the brain–gut axis, and the impact of psychosocial factors (Chey et al. 2015). Chronic stress not only manifests as depressive and anxiety‐like behaviors but may also trigger behavioral disorders through stress‐induced immune activation. The gut–brain–microbiota axis is increasingly recognized as a potential therapeutic target for stress‐related behavioral disorders (Westfall et al. 2021). These findings underscore the crucial roles of immunity, gut microbiota, and inflammation in both IBS and GAD. In recent years, the application of modern omics technologies, such as metabolomics, has opened up novel angles on the mechanisms of these diseases (Bongiorno et al. 2022). In the pre‐analysis, we failed to identify that factors such as immune cells and gut microbiota play a mediating role in the pathogenesis of GAD caused by IBS. Therefore, we rethought the relationship between these factors and IBS/GAD. Building on this foundation, we employed MR analysis to systematically investigate the causal relationships of immune cells, plasma metabolites, inflammatory factors, and gut microbiota with IBS and GAD. Our study reveals the intricate connections between these factors and the two diseases, offering new perspectives on their pathogenesis. Through MR analysis, we have further confirmed that IBS is a risk factor for GAD (OR = 1.328). This finding aligns with other MR studies that have revealed a significant causal relationship between IBS and major depressive disorder (MDD) (OR = 1.328) as well as anxiety (OR = 1.0611) (S. Zhou et al. 2025).

Mounting evidence underscores how pivotal gut microbiota are to the emergence and course of various diseases. Through comprehensive analysis, we have revealed 35 gut bacterial taxa with a causal relationship to GAD and 25 taxa associated with IBS. Notably, “*Bifidobacterium*” exerted a protective effect against GAD (OR = 0.859), a finding corroborated by multiple studies. Probiotics, such as *Lactobacillus* and *Bifidobacterium*, have been proven to be highly effective in preventing and treating psychiatric disorders like anxiety and depression. They achieve this by modulating neurotransmitter metabolism, enhancing the function of the HPA axis, and upregulating the expression of brain‐derived neurotrophic factor and tyrosine kinase receptor B (Xiong et al. 2023). Additionally, *Lactobacillus* and *Bifidobacterium* can promote the production of gamma‐aminobutyric acid (GABA), a key neurotransmitter that helps regulate anxiety and stress responses (Nikel et al. 2025). Numerous studies have demonstrated that certain *Bifidobacterium* strains can reduce inflammation by preventing NF‐κB activation and lipopolysaccharide synthesis, lowering IL‐1β levels, and controlling immunological balance and inflammatory reactions (J. Chen et al. 2021). Clinical studies have further demonstrated that after 4 weeks of supplementation with probiotics, prebiotics, plant extracts, and nutrients, the abundance of *Lactobacillus* and *Bifidobacterium* significantly increased, thereby positively impacting mental health (Talbott et al. 2018). Moreover, we found that “*Odoribacter*” was protective factor for IBS (OR = 0.949). *Odoribacter* is essential for preserving intestinal health, and its reduced abundance is closely linked to inflammatory bowel disease (IBD) (S. F. Lima et al. 2022). Certain strains of *Odoribacter*, such as *O. splanchnicus* and *O. laneus* , can decrease inflammation and increase glucose tolerance by lowering gut succinate and generating outer membrane vesicles (Hiippala et al. 2020; Huber‐Ruano et al. 2022). These results unveil that *Odoribacter* may have a beneficial regulatory effect on the occurrence and progression of IBS. Collectively, these findings indicate that modulating the composition and function of the gut microbiota may offer novel therapeutic avenues for IBS and GAD.

Regarding inflammatory factors, we revealed 6 inflammatory factors associated with GAD and 7 with a causal relationship to IBS. Among them, “stem cell factor (SCF)” was a risk factor for IBS (OR = 1.032), which is supported by relevant studies. Evidence has shown that SCF expression is elevated in IBS patients (Yang et al. 2015). The SCF/c‐Kit system, which is essential for the survival and function of neural crest‐derived cells, plays a significant role in visceral perception, smooth muscle contraction, and inflammatory responses (Jin et al. 2013; Liu et al. 2013; Y. G. Sun et al. 2009). The SCF/c‐Kit axis may adversely affect pain perception, inflammatory responses, and bowel function by enhancing sensitivity to gastrointestinal hormones (Chai et al. 2017). The high visceral sensitivity, abnormal gut contraction intensity, and low‐grade inflammation that IBS patients experience may be explained by disruption of the neuroendocrine–immune network brought on by changes in the SCF/c‐Kit system, indicating that this system may be a promising target for IBS intervention (Eshraghian and Eshraghian 2011; Y. L. Zhou et al. 2012). Additionally, “fractalkine (FKN)” was a protective factor for GAD (OR = 0.870). Depending on its concentration, solubility, peptide size, age of microglia, and the neurocircuit microenvironment, FKN can cause either pro‐ or anti‐inflammatory reactions in microglia (Finneran et al. 2023; Morganti et al. 2012; Nash et al. 2015; Subbarayan et al. 2022). Under physiological conditions, disruption of FKN signaling may lead to elevated IL‐1β levels, thereby impairing cognitive function and synaptic plasticity (J. T. Rogers et al. 2011). These results reveal fresh perspectives on the pathophysiological processes that underlie GAD and IBS.

The immune system is a key regulator of both gut function and emotional responses. There is growing evidence that CD4^+^ T cells contribute an essential part in the onset and progression of chronic intestinal inflammation. IL‐1β and IL‐17, two cytokines linked to CD4^+^ T cells, are markedly elevated in the inflammatory mucosa of IBD patients (Zenewicz et al. 2009). Moreover, CD4^+^ T cells also contribute to visceral pain and hypersensitivity. Colon mucosal biopsies from individuals with IBS reveal a substantial increase in CD4^+^ T cell infiltration in the lamina propria (Spiller et al. 2000). Our MR analysis further indicates that “CD4^+^ T cell Absolute Count” was a risk factor for IBS (OR = 1.035), potentially driving its development through the production of pro‐inflammatory factors. Additionally, our MR analysis revealed “CD27 on unswitched memory B cell” as a risk factor for GAD (OR = 1.046). This finding is corroborated by another MR study reporting an association between CD27 on unswitched memory B cells and depression (OR = 1.015) (H. Xue et al. 2024). CD27 is predominantly expressed on B cells and natural killer (NK) cells (Millan et al. 2019; Odendahl et al. 2000), while its ligand, CD70, is a transmembrane glycoprotein primarily found on B cells and mature dendritic cells (Flieswasser et al. 2022). The interaction between CD27 and CD70 triggers intracellular signaling pathways, promoting cell activation, proliferation, effector function, and survival. It is also involved in the activation, proliferation, and differentiation of T cells and B cells (Gong et al. 2023; van de Ven and Borst 2015; Wajant 2016). However, the specific mechanisms by which CD27 contributes to GAD remains to be elucidated through further investigation.

The high sensitivity of metabolomics allows for the detection of subtle changes in biological pathways, thereby enhancing our understanding of various physiological states and disease mechanisms (Johnson et al. 2016). Our research revealed the “glutamine to alanine ratio” as a protective factor for IBS (OR = 0.966). Glutamine is a vital nutrient in the small intestinal mucosa, providing energy for metabolism, regulating cell proliferation, and participating in the repair and maintenance of intestinal barrier function (Young and Ajami 2001). It is considered a key nutrient for treating “leaky gut syndrome”, because it is the preferred energy source for enterocytes and colonic cells (DeMarco et al. 2003). Low serum glutamine levels are closely associated with intestinal barrier disruption, inflammation, and diarrhea in children (Guerrant et al. 2008; N. L. Lima et al. 2007). Clinical studies have demonstrated that glutamine supplements can significantly improve intestinal barrier function in patients under high stress and those receiving total parenteral nutrition (TPN). Moreover, enteral and parenteral diets supplemented with glutamine can greatly improve intestinal architecture and function (Fujita and Sakurai 1995; Li et al. 1994). In IBS‐D patients with increased intestinal permeability, oral glutamine supplements can significantly and safely improve all major IBS‐related symptoms (Q. Zhou et al. 2019). Additionally, elevated levels of alanine have been detected in the fecal samples of IBS patients (Ponnusamy et al. 2011), and improved alanine metabolism may reflect the levels of alanine in the gut, thereby alleviating IBS symptoms (Y. Y. Sun et al. 2018). These findings suggest that modulating the glutamine to alanine ratio could have a positive impact on IBS. Furthermore, through MR analysis, we discovered that “kynurenine (KYN) levels” was a risk factor for GAD (OR = 1.110). A growing body of evidence indicates the regulatory role of the immune–KYN pathway in anxiety. Tryptophan catabolites in the KYN pathway become imbalanced under stress or inflammatory conditions, leading to deficiencies in serotonin and melatonin, which in turn exacerbate anxiety responses (Kim and Jeon 2018), consistent with our findings. These discoveries provide novel angles on the part of metabolites in the pathogenesis of IBS and GAD.

Mediation analysis further elucidated the mechanisms through which immune cells (CD25 on resting CD4 regulatory T cells), gut microbiota (*Bilophila*), and metabolites (levels of 10‐heptadecenoate [17:1n7] and 1‐palmitoyl‐2‐linoleoyl‐gpc [16:0/18:2]) influence GAD by modulating IBS. These results indicate that IBS can regulate the effects of immune cells, intestinal microbiota, and plasma metabolites on GAD.

Although this study identified multiple factors affecting IBS and GAD and demonstrated that IBS, as a mediator, might influence the development of GAD, there are still some limitations. First, the study's conclusions may not be applicable to other races or populations, as the majority of the data were derived from GWAS of individuals of European descent. Second, the lack of longitudinal data limits the ability to fully capture the dynamic changes of these factors over time. Additionally, the absence of detailed subgroup analyses or stratification by population subgroups restricts the generalizability of the study findings. This study used unadjusted *p*‐values as the screening criteria. Although this is helpful for identifying potential biological signals, it may increase the risk of false positives. These findings need to be further verified through experiments in the future. Moreover, although mediation analysis has revealed the mediating role of IBS in the relationship between multiple factors and GAD, the specific functions of some mediating variables remain unclear and should be interpreted with caution.

## 5 Conclusion

To our knowledge, this is the first comprehensive evaluation of the causal links between immune cells, plasma metabolites, inflammatory factors, and gut microbiota in IBS and GAD. Through mediation analysis, we have further confirmed the complex links between these factors and the two diseases, providing a new theoretical basis for understanding their pathogenesis.

## Author Contributions

**Weili Yang:** conceptualization (equal), data curation (lead), formal analysis (supporting), investigation (lead), methodology (lead), project administration (lead), writing – original draft (lead), writing – review and editing (equal). **Zebo Jia:** conceptualization (lead), funding acquisition (lead), project administration (equal), resources (lead), supervision (lead), writing – review and editing (lead). **Yongxi Wang:** conceptualization (equal), formal analysis (equal), project administration (supporting), resources (supporting). **Shasha Wang:** conceptualization (equal), formal analysis (equal), methodology (supporting), writing – original draft (supporting). **Jun Che:** conceptualization (supporting), data curation (equal), investigation (supporting), methodology (supporting), writing – review and editing (equal). **Hongbing Zhai:** conceptualization (supporting), formal analysis (equal), resources (equal), validation (equal). **Xin Wang:** formal analysis (supporting), methodology (supporting), project administration (supporting), validation (equal). **Yafeng Yang:** investigation (equal), software (supporting), validation (supporting).

## Funding

This research was funded by the Science and Technology Bureau of Xianyang City (grant number: L2024‐ZDYF‐ZDYF‐SF‐0015).

## Ethics Statement

The authors have nothing to report.

## Consent

The authors have nothing to report.

## Conflicts of Interest

The authors declare no conflicts of interest.

## Supporting information


**Figure S1:** Results of Mendelian randomization (MR) analysis of irritable bowel syndrome (IBS) on generalized anxiety disorder (GAD). CI, confidence interval; nsnp, number of single nucleotide polymorphism; pleio_P, pleiotropy *p‐*value; pval, *p‐*value; or, odds ratio.

**Table S1:** STROBE‐MR‐checklist‐fillable.

**Table S2:** Screening results of single nucleotide polymorphisms (SNPs) with irritable bowel syndrome (IBS) and generalized anxiety disorder (GAD).

**Table S3:** Screening results of SNPs with immune cell characteristics with IBS and GAD.

**Table S4:** Screening results of SNPs with plasma metabolites with IBS and GAD.

**Table S5:** Screening results of SNPs with gut microbiota with IBS and GAD.

**Table S6:** Screening results of SNPs with inflammatory factors with IBS and GAD.

**Table S7:** The causal relationship between IBS and GAD.

**Table S8:** Heterogeneity and horizontal pleiotropy tests for Mendelian randomization (MR) analysis.

## Data Availability

The data analyzed in this study were retrieved from the GWAS catalog (https://www.ebi.ac.uk/gwas/), IEU OpenGWAS project (https://gwas.mrcieu.ac.uk/), and FinnGen R12 database (https://r12.finngen.fi/).
