# Supplementary figures and images for: The Association Between Irritable Bowel Syndrome and Generalized Anxiety Disorder and Influencing Factors: A Mediation Mendelian Randomization Study
Source: Food Sci Nutr. 2026 Feb 10;14(2):e71525. doi: 10.1002/fsn3.71525 (PMC12887448; doi:10.1002/fsn3.71525)

**
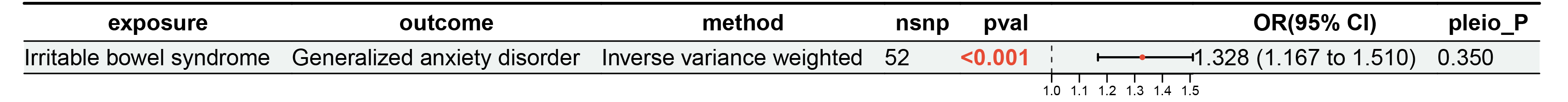
**

Supplement: Supplementary file 1 — Figure S1: Results of Mendelian randomization (MR) analysis of irritable bowel syndrome (IBS) on generalized anxiety disorder (GAD). CI, confidence interval; nsnp, number of single nucleotide polymorphism; pleio_P, pleiotropy p‐value; pval, p‐value; or, odds ratio. [file FSN3-14-e71525-s007.docx]
