# Supplementary material for: The Association Between Irritable Bowel Syndrome and Generalized Anxiety Disorder and Influencing Factors: A Mediation Mendelian Randomization Study
Source: Food Sci Nutr. 2026 Feb 10;14(2):e71525. doi: 10.1002/fsn3.71525 (PMC12887448; doi:10.1002/fsn3.71525)
Supplement: Supplementary file 2 — Table S1: STROBE‐MR‐checklist‐fillable. [file FSN3-14-e71525-s002.docx]

**STROBE-MR checklist of recommended items to address in reports of Mendelian randomization studies**^1^ ^2^

| **Item No.** | **Section** | **Checklist item** | **Page No.** | **Relevant text from manuscript** |
| --- | --- | --- | --- | --- |
| 1 | **TITLE and ABSTRACT** | Indicate Mendelian randomization (MR) as the study’s design in the title and/or the abstract if that is a main purpose of the study | 1 | The Association Between Irritable Bowel Syndrome and Generalized Anxiety Disorder and Influencing Factors: A Mediation Mendelian Randomization Study |
|  | **INTRODUCTION** |  |  |  |
| 2 | **Background** | Explain the scientific background and rationale for the reported study. What is the exposure? Is a potential causal relationship between exposure and outcome plausible? Justify why MR is a helpful method to address the study question | 4 | While prior research has examined the contributions of immune cells, plasma metabolites, inflammatory factors, and gut microbiota to IBS/GAD, the precise interrelationships remain elusive. Given that immune cells, plasma metabolites, inflammatory factors and the gut microbiota all play significant roles in the pathogenesis of IBS and GAD. Therefore, this study aims to explore the influence of factors such as immune cells and plasma metabolites on IBS/GAD. In addition, IBS patients also exhibit symptoms of GAD. Therefore, we also further explored the mediating role of IBS. Genetic epidemiology has made considerable use of Mendelian randomization (MR), a method of causal inference based on genetic variation (Sekula, Del Greco, Pattaro, & Köttgen, 2016). MR offers advantages over conventional observational studies by utilizing genetic variants as instrumental variables (IVs) to mitigate confounding, thereby bolstering the robustness of causal inferences (Sanderson et al., 2022). |
| 3 | **Objectives** | State specific objectives clearly, including pre-specified causal hypotheses (if any). State that MR is a method that, under specific assumptions, intends to estimate causal effects | 4 | Therefore, this study aims to separately explore the influence of factors such as immune cells and plasma metabolites on IBS/GAD. |
|  | **METHODS** |  |  |  |
| 4 | **Study design and data sources** | Present key elements of the study design early in the article. Consider including a table listing sources of data for all phases of the study. For each data source contributing to the analysis, describe the following: |  |  |
|  | a) | Setting: Describe the study design and the underlying population, if possible. Describe the setting, locations, and relevant dates, including periods of recruitment, exposure, follow-up, and data collection, when available. | 4-5 | The genome-wide association study (GWAS) data on plasma metabolites, immune cell characteristics, and gut microbiota could be obtained from the GWAS catalog (https://www.ebi.ac.uk/gwas/). The Canadian Longitudinal Study on Aging (CLSA) cohort provided GWAS data on 1,400 plasma metabolites, covering 8,091 individuals (GCST90199621 to GCST90201020) (Y. Chen et al., 2023). The GWAS data on 731 immune cell characteristics (GCST0001391 to GCST0002121) involved 3,757 non-overlapping European individuals (Orrù et al., 2020). The data on gut microbiota were derived from the Dutch Microbiome Project, which included 7,738 participants and measured 207 microbial taxa and 205 metabolic pathways (GCST90027446 to GCST90027857) (Lopera-Maya et al., 2022). A study used the Olink Target Inflammation panel to measure 91 inflammation-related circulating proteins, involving 11 cohorts and 14,824 European participants (Zhao et al., 2023). The data on IBS were downloaded from the IEU OpenGWAS project (https://gwas.mrcieu.ac.uk/). This ebi-a-GCsT90016564 dataset included 53,400 cases and 433,201 control samples of European ancestry, with a total of 9,739,966 single nucleotide polymorphisms (SNPs). The data on GAD (finngen_R12_F5_GAD) were obtained from FinnGen R12 (https://r12.finngen.fi/), including 7,148 cases and 444,414 European control samples (Kurki et al., 2023). |
|  | b) | Participants: Give the eligibility criteria, and the sources and methods of selection of participants. Report the sample size, and whether any power or sample size calculations were carried out prior to the main analysis | 4-5 | The genome-wide association study (GWAS) data on plasma metabolites, immune cell characteristics, and gut microbiota could be obtained from the GWAS catalog (https://www.ebi.ac.uk/gwas/). The Canadian Longitudinal Study on Aging (CLSA) cohort provided GWAS data on 1,400 plasma metabolites, covering 8,091 individuals (GCST90199621 to GCST90201020) (Y. Chen et al., 2023). The GWAS data on 731 immune cell characteristics (GCST0001391 to GCST0002121) involved 3,757 non-overlapping European individuals (Orrù et al., 2020). The data on gut microbiota were derived from the Dutch Microbiome Project, which included 7,738 participants and measured 207 microbial taxa and 205 metabolic pathways (GCST90027446 to GCST90027857) (Lopera-Maya et al., 2022). A study used the Olink Target Inflammation panel to measure 91 inflammation-related circulating proteins, involving 11 cohorts and 14,824 European participants (Zhao et al., 2023). The data on IBS were downloaded from the IEU OpenGWAS project (https://gwas.mrcieu.ac.uk/). This ebi-a-GCsT90016564 dataset included 53,400 cases and 433,201 control samples of European ancestry, with a total of 9,739,966 single nucleotide polymorphisms (SNPs). The data on GAD (finngen_R12_F5_GAD) were obtained from FinnGen R12 (https://r12.finngen.fi/), including 7,148 cases and 444,414 European control samples (Kurki et al., 2023). |
|  | c) | Describe measurement, quality control and selection of genetic variants | 5-6 | To maximize the utility of the IVs, the following selection criteria were applied: When the exposure factors were immune cell characteristics, plasma metabolites, inflammatory factors, and gut microbiota, SNPs with P < 1*10-5 were chosen as IVs; whereas for IBS or GAD exposures, a more stringent criterion of P < 5*10-6 was adopted. The ieugwasr package (v 1.0.0) (Fan, Lu, Gan, & Lu, 2024) was used to exclude SNPs with linkage disequilibrium, with parameters set at r² = 0.001 and kb = 10000. Additionally, SNPs that were substantially linked to the exposure or result (P < 1*10-5) were excluded via GWAS catalog to avoid potential horizontal pleiotropy. To assess the strength of each SNP as an IV, the F-statistic was worked out, and SNPs with F-statistic less than 10 were excluded. Subsequently, the harmonise_data function was utilized to integrate exposure and outcome data, ensuring consistent SNP effect directions. Finally, the steiger_filtering function was solicited to detect the direction of association, retaining only SNPs with associations from exposure to outcome. |
|  | d) | For each exposure, outcome, and other relevant variables, describe methods of assessment and diagnostic criteria for diseases | 6 | The study first employed MR analysis to systematically analyze the causal connection between immune cell characteristics, plasma metabolites, inflammatory factors, and gut microbiota with IBS and GAD respectively. Additionally, the mediating effects of IBS on the associations between these multiple factors and GAD were thoroughly analyzed. In terms of analytical methods, the inverse variance weighted (IVW) model (Ding et al., 2023) was resorted to as the cardinal tool. To verify the robustness of the results, several supplementary methods were also employed, including MR-Egger (Burgess & Thompson, 2017), weighted median (Bowden, Davey Smith, Haycock, & Burgess, 2016), simple mode (Zeng, Cao, & Yang, 2023), and weighted mode (Zeng et al., 2023), to estimate causal effects from multiple perspectives. |
|  | e) | Provide details of ethics committee approval and participant informed consent, if relevant | NA | NA |
| 5 | **Assumptions** | Explicitly state the three core IV assumptions for the main analysis (relevance, independence and exclusion restriction) as well assumptions for any additional or sensitivity analysis | 5 | The entire study was based on three fundamental assumptions: (1) There should be a significant connection between the exposure and the IVs chosen. (2) IVs ought to be unaffected by confounding variables. (3) IVs must only influence the result by exposure. |
| 6 | **Statistical methods: main analysis** | Describe statistical methods and statistics used |  |  |
|  | a) | Describe how quantitative variables were handled in the analyses (i.e., scale, units, model) | NA | NA |
|  | b) | Describe how genetic variants were handled in the analyses and, if applicable, how their weights were selected | 5-6 | To maximize the utility of the IVs, the following selection criteria were applied: When the exposure factors were immune cell characteristics, plasma metabolites, inflammatory factors, and gut microbiota, SNPs with P < 1*10-5 were chosen as IVs; whereas for IBS or GAD exposures, a more stringent criterion of P < 5*10-6 was adopted. The ieugwasr package (v 1.0.0) (Fan, Lu, Gan, & Lu, 2024) was used to exclude SNPs with linkage disequilibrium, with parameters set at r² = 0.001 and kb = 10000. Additionally, SNPs that were substantially linked to the exposure or result (P < 1*10-5) were excluded via GWAS catalog to avoid potential horizontal pleiotropy. To assess the strength of each SNP as an IV, the F-statistic was worked out, and SNPs with F-statistic less than 10 were excluded. Subsequently, the harmonise_data function was utilized to integrate exposure and outcome data, ensuring consistent SNP effect directions. Finally, the steiger_filtering function was solicited to detect the direction of association, retaining only SNPs with associations from exposure to outcome. |
|  | c) | Describe the MR estimator (e.g. two-stage least squares, Wald ratio) and related statistics. Detail the included covariates and, in case of two-sample MR, whether the same covariate set was used for adjustment in the two samples | 6-7 | The MRPRESSO tool was operated to detect and correct for pleiotropy by identifying and removing potential outliers with P < 0.05, thereby enhancing the stoutness of the analysis results. Additionally, to ascertain whether there truly was horizontal pleiotropy throughout IVs and the result, the MR-Egger intercept (P > 0.05) was taken into account. To quantify the degree of heterogeneity, the P-value derived from the Cochrane Q statistic was reckoned. |
|  | d) | Explain how missing data were addressed | NA | NA |
|  | e) | If applicable, indicate how multiple testing was addressed |  |  |
| 7 | **Assessment of assumptions** | Describe any methods or prior knowledge used to assess the assumptions or justify their validity | NA | NA |
| 8 | **Sensitivity analyses and additional analyses** | Describe any sensitivity analyses or additional analyses performed (e.g. comparison of effect estimates from different approaches, independent replication, bias analytic techniques, validation of instruments, simulations) | 6-7 | The MRPRESSO tool was operated to detect and correct for pleiotropy by identifying and removing potential outliers with P < 0.05, thereby enhancing the stoutness of the analysis results. Additionally, to ascertain whether there truly was horizontal pleiotropy throughout IVs and the result, the MR-Egger intercept (P > 0.05) was taken into account. To quantify the degree of heterogeneity, the P-value derived from the Cochrane Q statistic was reckoned. Leave-one-out was also executed to evaluate whether the IVW estimate was overly influenced by individual SNPs (Hemani, Bowden, & Davey Smith, 2018). |
| 9 | **Software and pre-registration** |  |  |  |
|  | a) | Name statistical software and package(s), including version and settings used | 6 | All analyses were primarily based on the TwoSampleMR (v 0.6.0) (Hemani, Zheng, et al., 2018) and MRPRESSO packages (v 1.0) (Zhu, Hu, & Fan, 2022). |
|  | b) | State whether the study protocol and details were pre-registered (as well as when and where) | NA | NA |
|  | **RESULTS** |  |  |  |
| 10 | **Descriptive data** |  |  |  |
|  | a) | Report the numbers of individuals at each stage of included studies and reasons for exclusion. Consider use of a flow diagram | NA | NA |
|  | b) | Report summary statistics for phenotypic exposure(s), outcome(s), and other relevant variables (e.g. means, SDs, proportions) | 7 | In the genetic studies of IBS and GAD, a total of 52 SNPs were included, with F-values spanning from 20.863 to 36.993. In the reverse MR analysis, 18 SNPs were selected, with F-values varying from 20.957 to 28.681 (Supplementary Table 2). For the study of immune cell characteristics and IBS, 17,358 SNPs were used, while for the study with GAD, 17,564 SNPs were selected, with F-values spanning from 19.548 to 2435.818 (Supplementary Table 3). In the analysis of plasma metabolites and IBS, 32,868 SNPs were obtained; in the analysis with GAD, 33,335 SNPs were obtained, with F-values fluctuating from 19.511 to 5309.700 (Supplementary Table 4). In the investigation of gut microbiota and IBS, 4,036 SNPs were obtained, while in the study with GAD, 3,910 SNPs were obtained, with F-values fluctuating from 19.512 to 61.116 (Supplementary Table 5). Finally, in the study of inflammatory factors and IBS, 2,503 SNPs were selected, while in the study with GAD, 2,345 SNPs were obtained, with F-values varying from 19.513 to 1477.144 (Supplementary Table 6). These results demonstrate that the selected SNPs have strong statistical power, providing a reliable basis for subsequent causal relationship analyses. |
|  | c) | If the data sources include meta-analyses of previous studies, provide the assessments of heterogeneity across these studies | NA | NA |
|  | d) | For two-sample MR:  i.  Provide justification of the similarity of the genetic variant-exposure associations between the exposure and outcome samples  ii.  Provide information on the number of individuals who overlap between the exposure and outcome studies | NA | NA |
| 11 | **Main results** |  |  |  |
|  | a) | Report the associations between genetic variant and exposure, and between genetic variant and outcome, preferably on an interpretable scale | 10 | To further investigate the causal association between IBS and GAD, the MR method was employed. The IVW results indicated that IBS was risk factor for GAD [odds ratio (OR) = 1.328; 95% confidence interval (CI) = 1.167-1.510; P < 0.001, false discovery rate (FDR) < 0.001] (Supplementary Figure 1) (Supplementary Table 7). |
|  | b) | Report MR estimates of the relationship between exposure and outcome, and the measures of uncertainty from the MR analysis, on an interpretable scale, such as odds ratio or relative risk per SD difference | 8 | After traversing the causal relationships between 207 microbial taxa and 205 pathways and IBS, 25 significant causal relationships were revealed, with 10 considered protective factors and 15 as risk factors (Figure 1). Specifically, the "superpathway of L-aspartate and L-asparagine biosynthesis" (OR = 0.901; 95%CI = 0.848-0.957; P < 0.001) and "Eggerthella" (OR = 0.946; 95%CI = 0.910-0.984; P = 0.005) were found to have protective effects on IBS. |
|  | c) | If relevant, consider translating estimates of relative risk into absolute risk for a meaningful time period | NA | NA |
|  | d) | Consider plots to visualize results (e.g. forest plot, scatterplot of associations between genetic variants and outcome versus between genetic variants and exposure) | 10 | Figure 1 |
| 12 | **Assessment of assumptions** |  |  |  |
|  | a) | Report the assessment of the validity of the assumptions | 11 | To ensure the robustness and reliability of the results, a variety of methods were employed. MR-Egger and MR-PRESSO failed to pinpoint any hypothetical pleiotropy (Supplementary Table 8). |
|  | b) | Report any additional statistics (e.g., assessments of heterogeneity across genetic variants, such as *I^2^*, Q statistic or E-value) | 11 | Faced with heterogeneity, we settled on random-effects model prior to the study to avoid bias caused by heterogeneity (Supplementary Table 8). |
| 13 | **Sensitivity analyses and additional analyses** |  |  |  |
|  | a) | Report any sensitivity analyses to assess the robustness of the main results to violations of the assumptions | 11 | To ensure the robustness and reliability of the results, a variety of methods were employed. MR-Egger and MR-PRESSO failed to pinpoint any hypothetical pleiotropy (Supplementary Table 8). Faced with heterogeneity, we settled on random-effects model prior to the study to avoid bias caused by heterogeneity (Supplementary Table 8). By sequentially excluding individual SNPs and re-analyzing the remaining SNPs, we encountered that outcomes were not dramatically modified by the removal of a single SNP. These findings provided strong support for the results. |
|  | b) | Report results from other sensitivity analyses or additional analyses | NA | NA |
|  | c) | Report any assessment of direction of causal relationship (e.g., bidirectional MR) | 10 | However, the reverse MR analysis did not demonstrate a causal link between GAD and IBS. |
|  | d) | When relevant, report and compare with estimates from non-MR analyses | NA | NA |
|  | e) | Consider additional plots to visualize results (e.g., leave-one-out analyses) | 11 | By sequentially excluding individual SNPs and re-analyzing the remaining SNPs, we encountered that outcomes were not dramatically modified by the removal of a single SNP. |
|  | **DISCUSSION** |  |  |  |
| 14 | **Key results** | Summarize key results with reference to study objectives | 13 | Mounting evidence underscores how pivotal gut microbiota are to the emergence and course of various diseases. Through comprehensive analysis, we have revealed 35 gut bacterial taxa with a causal relationship to GAD and 25 taxa associated with IBS. |
| 15 | **Limitations** | Discuss limitations of the study, taking into account the validity of the IV assumptions, other sources of potential bias, and imprecision. Discuss both direction and magnitude of any potential bias and any efforts to address them | 16-17 | First, the study's conclusions may not be applicable to other races or populations, as the majority of the data were derived from GWAS of individuals of European descent. Second, the lack of longitudinal data limits the ability to fully capture the dynamic changes of these factors over time. Additionally, the absence of detailed subgroup analyses or stratification by population subgroups restricts the generalizability of the study findings. This study used unadjusted P-values as the screening criteria. Although this is helpful for identifying potential biological signals, it may increase the risk of false positives. These findings need to be further verified through experiments in the future. Moreover, although mediation analysis has revealed the mediating role of IBS in the relationship between multiple factors and GAD, the specific functions of some mediating variables remain unclear and should be interpreted with caution. |
| 16 | **Interpretation** |  |  |  |
|  | a) | Meaning: Give a cautious overall interpretation of results in the context of their limitations and in comparison with other studies | 16-17 | First, the study's conclusions may not be applicable to other races or populations, as the majority of the data were derived from GWAS of individuals of European descent. Second, the lack of longitudinal data limits the ability to fully capture the dynamic changes of these factors over time. Additionally, the absence of detailed subgroup analyses or stratification by population subgroups restricts the generalizability of the study findings. This study used unadjusted P-values as the screening criteria. Although this is helpful for identifying potential biological signals, it may increase the risk of false positives. These findings need to be further verified through experiments in the future. Moreover, although mediation analysis has revealed the mediating role of IBS in the relationship between multiple factors and GAD, the specific functions of some mediating variables remain unclear and should be interpreted with caution. |
|  | b) | Mechanism: Discuss underlying biological mechanisms that could drive a potential causal relationship between the investigated exposure and the outcome, and whether the gene-environment equivalence assumption is reasonable. Use causal language carefully, clarifying that IV estimates may provide causal effects only under certain assumptions | 13 | Moreover, we found that "Odoribacter" was protective factor for IBS (OR = 0.949). Odoribacter is essential for preserving intestinal health, and its reduced abundance is closely linked to inflammatory bowel disease (IBD) (S. F. Lima et al., 2022). Certain strains of Odoribacter, such as O. splanchnicus and O. laneus, can decrease inflammation and increase glucose tolerance by lowering gut succinate and generating outer membrane vesicles (Hiippala et al., 2020; Huber-Ruano et al., 2022). These results unveil that Odoribacter may have a beneficial regulatory effect on the occurrence and progression of IBS. |
|  | c) | Clinical relevance: Discuss whether the results have clinical or public policy relevance, and to what extent they inform effect sizes of possible interventions | 15-16 | Glutamine is a vital nutrient in the small intestinal mucosa, providing energy for metabolism, regulating cell proliferation, and participating in the repair and maintenance of intestinal barrier function (Young & Ajami, 2001). It is considered a key nutrient for treating "leaky gut syndrome", because it is the preferred energy source for enterocytes and colonic cells (DeMarco, Li, Thomas, West, & Neu, 2003). Low serum glutamine levels are closely associated with intestinal barrier disruption, inflammation, and diarrhea in children (Guerrant, Oriá, Moore, Oriá, & Lima, 2008; N. L. Lima et al., 2007). Clinical studies have demonstrated that glutamine supplements can significantly improve intestinal barrier function in patients under high stress and those receiving total parenteral nutrition (TPN). Moreover, enteral and parenteral diets supplemented with glutamine can greatly improve intestinal architecture and function (Fujita & Sakurai, 1995; Li, Langkamp-Henken, Suzuki, & Stahlgren, 1994). In IBS-D patients with increased intestinal permeability, oral glutamine supplements can significantly and safely improve all major IBS-related symptoms (Q. Zhou et al., 2019). Additionally, elevated levels of alanine have been detected in the fecal samples of IBS patients (Ponnusamy, Choi, Kim, Lee, & Lee, 2011), and improved alanine metabolism may reflect the levels of alanine in the gut, thereby alleviating IBS symptoms (Y. Y. Sun et al., 2018). These findings suggest that modulating the glutamine to alanine ratio could have a positive impact on IBS. |
| 17 | **Generalizability** | Discuss the generalizability of the study results (a) to other populations, (b) across other exposure periods/timings, and (c) across other levels of exposure | 16 | First, the study's conclusions may not be applicable to other races or populations, as the majority of the data were derived from GWAS of individuals of European descent. Second, the lack of longitudinal data limits the ability to fully capture the dynamic changes of these factors over time. Additionally, the absence of detailed subgroup analyses or stratification by population subgroups restricts the generalizability of the study findings. |
|  | **OTHER INFORMATION** |  |  |  |
| 18 | **Funding** | Describe sources of funding and the role of funders in the present study and, if applicable, sources of funding for the databases and original study or studies on which the present study is based | 18 | This research was funded by the Xianyang Science and Technology Bureau (grant number: L2024-ZDYF-ZDYF-SF-0015). |
| 19 | **Data and data sharing** | Provide the data used to perform all analyses or report where and how the data can be accessed, and reference these sources in the article. Provide the statistical code needed to reproduce the results in the article, or report whether the code is publicly accessible and if so, where | 18 | The data analyzed in this study were retrieved from the GWAS catalog (https://www.ebi.ac.uk/gwas/), IEU OpenGWAS project (https://gwas.mrcieu.ac.uk/), and FinnGen R12 database (https://r12.finngen.fi/). |
| 20 | **Conflicts of Interest** | All authors should declare all potential conflicts of interest | 18 | The authors report no conflict of interest concerning the materials or methods used in this study or the findings specified in this paper. |

This checklist is copyrighted by the Equator Network under the Creative Commons Attribution 3.0 Unported (CC BY 3.0) license.

1. Skrivankova VW, Richmond RC, Woolf BAR, Yarmolinsky J, Davies NM, Swanson SA, et al. Strengthening the Reporting of Observational Studies in Epidemiology using Mendelian Randomization (STROBE-MR) Statement. JAMA. 2021;under review.

2. Skrivankova VW, Richmond RC, Woolf BAR, Davies NM, Swanson SA, VanderWeele TJ, et al. Strengthening the Reporting of Observational Studies in Epidemiology using Mendelian Randomisation (STROBE-MR): Explanation and Elaboration. BMJ. 2021;375:n2233.
